# Priority mental, neurological and substance use disorders in rural Kenya: Traditional health practitioners’ and primary health care workers’ perspectives

**DOI:** 10.1371/journal.pone.0220034

**Published:** 2019-07-23

**Authors:** Mary A. Bitta, Symon M. Kariuki, Joseph Gona, Amina Abubakar, Charles R. J. C. Newton

**Affiliations:** 1 Tropical Neurosciences, KEMRI/Wellcome Trust Research Programme, Centre for Geographic Medicine Research (Coast), Kilifi, Kenya; 2 Department of Psychiatry, University of Oxford, Oxford, United Kingdom; 3 Aga Khan University, Nairobi, Kenya; Addis Ababa University / King's College London, ETHIOPIA

## Abstract

**Background:**

Over 75% of people with mental neurological and substance use disorders (MNSD) live in low and middle-income countries with limited access to specialized care. The World Health Organization’s Mental Health Gap Action Program (mhGAP) aims to address the human resource gap but it requires contextualization.

**Aims:**

We conducted a qualitative study in rural coastal Kenya to explore the local terms, perceived causes and management modalities of priority MNSD listed in the mhGAP, to inform implementation in this setting.

**Methods:**

We conducted 8 focus group discussions with primary health care providers and traditional health practitioners and used the framework method to conduct thematic analysis. We identified local terms, perceived causes and treatment options for MNSD. We also explored possibilities for collaboration between the traditional health practitioners and primary health care providers.

**Results:**

We found local terms for depression, psychoses, epilepsy, disorders due to substance use and self-harm/ suicide but none for dementia. Child and adolescent mental and behavioral problems were not regarded as MNSD but consequences of poor parenting. Self-harm/suicide was recognized in the context of other MNSD. Causes of MNSD were broadly either biological or supernatural. Treatment options were dependent on perceived cause of illness. Most traditional health practitioners were willing to collaborate with primary health care providers mainly through referring cases. Primary health care providers were unwilling to collaborate with traditional health practitioners because they perceived them to contribute to worsening of patients’ prognoses.

**Conclusions:**

Local terms and management modalities are available for some priority MNSD in this setting. Community level case detection and referral may be hindered by lack of collaboration between traditional health practitioners and primary health care providers. There is need for training on the recognition and management of all priority MNSD.

## Introduction

Low and middle-income countries (LMIC) account for majority of the disability-adjusted life years associated with mental, neurological and substance use disorders (MNSD) [1], but health care resources in these regions are constrained. Although the World Health Organization (WHO) recommends integration of mental health care into primary health care [2] this may be challenging in many LMIC, where formal health care systems are weak. In many LMIC, the ratio of health care workers per 1000 population is less than one (3) and this is further complicated by psychosocial issues surrounding mental health care, where MNSD are perceived from a cultural perspective [3–5]. This and other challenges such as poverty and cultural beliefs often lead to communities preferring alternative forms of healthcare to biomedical treatment [6–8].

Optimizing treatment and care for people with MNSD in LMIC requires utilization of all available resources within a health care system. This includes both formal resources such as primary health care providers and informal resources such as traditional health practitioners and faith healers, who have been shown to form part of the health care system in sub-Saharan Africa[9]. A recent study that geocoded all accessible hospitals in sub-Saharan Africa [10]showed a promising distribution of primary care facilities which could be utilised to increase coverage of mental health services through task sharing with non-specialist health care providers. However, successful task sharing will require investment in proper training of the health care providers as well as engaging alternative health care providers such as traditional health practitioners and faith healers. Studies have shown that traditional health practitioners are proximal to the community and are often accepted as medicine men who manage a wide range of illnesses including MNSD. Services from traditional health practitioners influence the time taken by clients to access formal health care services, with patients seeking treatment when their conditions are already severe[11] which leads to poor prognosis [9]. Therefore, effective management of MNSD, would not only need task sharing in primary health care facilities, but also inclusion of traditional health practitioners to enhance early case detection and referral.

The mental health Gap Action Program Intervention Guide (mhGAP-IG) is a guideline that was developed by the World Health Organization (WHO) in response to the growing burden of MNSD and the disproportionately low number of mental health specialists and facilities in many LMIC [2]. It contains seven priority conditions namely depression, psychoses, epilepsy, child and adolescent mental and behavioural disorders, dementia, disorders due to substance use and suicide/self-harm. The aim is to increase the capacity of WHO member states in managing MNSD by training non-specialists to detect and manage these illnesses. The mhGAP-IG has been contextualized and successfully used in several LMIC ([12–14]. In Kilifi Kenya, we are currently contextualizing the guideline for the setting. Contextualization of mhGAP in a rural area requires an understanding of the perceptions of MNSD by both formal and informal health care providers particularly primary health care providers and traditional healers. Previous studies on neurological conditions in Kilifi found that traditional health practitioners formed part of the pathway of care alongside primary health care providers [15]. Studies on the understanding of some priority mental disorders specifically depression [16]developmental disorders [17]and behavioural and emotional problems have been conducted in Kilifi. Some of these studies overemphasized on symptoms or presentations of these conditions rather than the local terms. For example, the study by Abubakar and colleagues that sought to understand the community perceptions of developmental and behavioural problems in children in rural Kenya, described the symptoms of these disorders based on caregiver reports but not local terms[18].Other studies were based on subpopulations e.g the study by Gona et al which was based on parents of children with autism spectrum disorders and professionals who manage the children and the study by Ssewanyanna et al which assessed socioecological determinants of substance use among adolescents [17, 19], yet other populations also experience challenges of mental health disorders.”. Other priority conditions such as dementia have not been previously studied in this setting. Additionally, data on the local understanding of most priority disorders in this setting are absent.

We postulate that successful implementation of the mhGAP-IG in this setting requires including traditional health practitioners in the implementation plan, specifically for case detection and referral. This is because studies on traditional healers’ involvement in the management of MNSD in Kenya have found that they are often preferred to primary health care providers because they provide more culturally acceptable explanations for diseases, are more easily accessible, they spend more time with clients and allow flexible modes of payments[20, 21]. To objectively contextualize the mhGAP, we need to understand the perspectives of both the traditional health practitioners and primary health care providers on MNSD to enable us plan for the best implementation strategy since both are in the pathway of care for MNSD in the study setting. Therefore, this study sought to establish which of the priority MNSD are commonly managed by health care providers and traditional health practitioners to inform implementation of the mhGAP-IG. We aimed to inform planning strategies that increase case detection by non-specialists as well as explore potential for collaboration between biomedical health care providers and alternative health care providers such as traditional healers.

## Methods

We conducted focus group discussions with traditional health practitioners and primary health care providers. We asked four main questions: (i) What are some of the common MNSD in the community, including their local terms? (ii) How do people with MNSD present? (iii) How do you manage these disorders? (iv)What are your perceptions about collaboration with traditional healers/primary health care providers?

### Study site

This study was conducted at Kilifi County on the coast of Kenya. Kilifi is one of the poorest regions of Kenya with a poverty level of approximately 71% (Kenya Commission on Revenue Allocation). The inhabitants of this area are predominantly of the Mijikenda ethnic group (80%) and the main languages spoken are Swahili, Kigiryama and Chonyi. About 70% of the inhabitants practice Christianity, 20% traditional religion and 10% Islam. The main economic activity is farming and the average per capita income for a household with typically both parents and six children is 1000 Kenyan shillings per month which is approximately 10$ (Kenya Population and Housing Census, 2009). Mental health services in Kilifi are scarce; there are no psychiatrists or psychologists in the county and there are only two mental health outpatient clinics for a population of approximately 1.2 million people. There are no mental health referral facilities and patients with mental illnesses are admitted to general medical wards based on availability of beds[22].

### Sampling and recruitment

Purposive sampling was used to select participants of this study. The study participants were primary health care providers and traditional healers. The sampling framework was designed to ensure maximum variation in terms of the age, sex, geographical locations and cadres of health care providers and traditional healers. Primary health care providers included nurses of different cadres and public health specialists selected from mapped healthcare facilities in the area. Nurses and public health officers were identified through the office of the nursing officer in charge of the hospital. We approached 31 nurses and 3 public health officers and although there were no refusals, 2 nurses, scheduled for the last FGD, were not able to participate because they arrived late when the discussions had been concluded. For this study we defined traditional health practitioners as persons who used long established methods passed down from one generation to another, such as exorcism or a combination of herbs and concoctions, to treat people suffering from various illnesses. Traditional health practitioners were identified from a database that had been established in a previous study[15] and through key informants. We approached 8 traditional health practitioners and identified an additional 23 through snowballing. Two traditional health practitioners died before participating in this study in what was reported as “complications related to advanced age” and one had hearing challenges and could therefore not participate, bringing the total sample size of traditional health practitioners to 28. We selected participants to represent the 7 constituencies of Kilifi County.

### Procedures

We carried out 8 focus group discussions with a total of 60 participants (28 traditional health practitioners and 32 health care providers). The FGD were conducted until saturation was reached whereby the were no new information and participants of different cardres and from different geographical locations were providing similar responses to questions. Each FGD had either 7 or 8 participants and the traditional health practitioners were interviewed separately from the primary health care providers. These discussions lasted approximately 1 hour each. All participants were either Swahili, English or Kigiryama speakers and were interviewed by either MB or FY who are fluent in both Kiswahili and English. Additionally, FY who is a native Kigiryama speaker recorded all the Kigiryama terms that were used in the discussion. The topic guide and probe questions used for the FGD have been provided as supporting materials in S1 File. All interviewers had tertiary level of education and had experience in conducting FGDs for previous studies. The FGDs were conducted between February 2018 and April 2018 and in total all 4 FGD for traditional health practitioners were administered in Kiswahili and 4 for primary health care providers in English. The questionnaire contained questions about the knowledge, beliefs and practices of the participants towards causes and management of the 7-priority MNSD (depression, psychoses, epilepsy, child and adolescent mental and behavioral disorders, dementia, disorders due to substance use and self-harm/suicide) listed in the mhGAP-IG. The interviews were audiotaped, and detailed notes were taken by MB, SK and FY. The audio data was first transcribed in the original language then all notes in Kiswahili or Kigiryama were translated to English. The interview questions were adopted from the mental health Gap Action Programme Intervention Guide (mhGAP-IG) implementation guidelines which recommend translation of the guidelines where necessary and an assessment of the available resources within a health system. Before asking questions about management for a specific condition, a vignette was presented describing the condition. These vignettes were adopted from a practical manual for non-specialist practitioners who manage patients with MNSD (18). An example of a vignette for psychosis is provided below:

“*Gambo*, *a 20-year-old university student*, *was brought* [to traditional healer/ primary health care provider] *because she locks herself in her room*. *Gambo used to be a good student but has failed her last exams*. *Her mother said that she would often spend hours staring into space*. *Sometimes she appeared to say things to herself in a low and inaudible voice as if she were talking to an imaginary person*. *Gambo was forced to come to the clinic/traditional healer by her parents*. *At first*, *she refused to talk to the nurse/traditional healer*. *After a while she admitted that she believed her parents and neighbours were plotting to kill her and that the devil was interfering with her mind*. *She also said that she did not see why she had been brought to the hospital/traditional healer*.*”*

### Data analysis

The framework method [23]was used to carry out thematic analysis of the transcripts. We used N-Vivo version 10 software (QSR International; http://www.qsrinternational.com) for data storage and management. Inductive coding was used to code the data and then thematic analysis was applied.

Data analysis was independently conducted by MB and JG followed by comparison and discussion of results. Where there were discrepancies between the results consensus was reached through a joint analysis.

Discussions were held, and consensus reached where there were conflicts between MB and JG. To maintain the anonymity of participants, all quotations used fictional name. The final themes were chosen after discussions between the study investigators. Initially, 17 codes were developed, and they were then grouped into the four main themes.

### Ethical considerations

This study was approved by the Scientific Ethical Review Unit at the Kenya Medical Research Institute under protocol number KEMRI/SERU/CGMR-C/O38/3260.

## Results

### Sociodemographic characteristics of study participants

Most of the study participants were male (66.7%). The age range of the traditional health practitioners was 31–75 years while that of primary health care providers was 26–44 years. The results of this study were presented in four broad themes namely local names of mental illness, perceived causes of MNSD, common symptoms of MNSD and management of MNSD.

### Local terms for common mental and neurological disorders

According to traditional healers, most people with mental disorders had psychosis as summed up by mama Rhoda, who was describing patients shown on a vignette with symptoms of psychosis: 6

‘*Yes*, *many*, *very many*, *those are the majority*.***’***

Participants from both groups referred to psychosis as madness. Both groups described epilepsy and dementia, which are neurological conditions, as mental illnesses. Among some traditional healers, names of physical illnesses were used to refer to neurological disorders for example tetanus was used interchangeably with epilepsy. Regarding epilepsy, Ayubu a traditional healer said:

‘*Mental illnesses are like madness and epilepsy*. *Epilepsy starts when a child is young*. *We sometimes call it epilepsy or tetanus*.*’*

Dementia was not perceived as a mental disorder by the health care providers because they thought of the main symptoms such as forgetfulness as a milestone of old age, but they thought it does not occur to everybody.

Health care providers described depression as a problem which interferes with somebody’s moods as summed up by Rudi:

‘*Yes*, *it’s a problem because depression can interfere with somebody’s moods*.*’*

For disorders due to substance use, participants described two categories of patients: (i) users of drugs such as heroin, cocaine and bhang, who were referred to as “Maunga” and (ii) people with alcohol use disorders, who were referred to as “drunkards”.

Substances which were commonly abused included tobacco and bhang. Palm wine, commonly referred to as ‘mnazi’ was the most commonly abused alcoholic drink. When asked about the types of substances used by patients they had encountered in their practice, one nurse said:

‘*There is bhang and tobacco*, *especially with young boys from the age of 18 to 21*.*’*

There were no local terms for dementia. The use of detailed symptomatology to describe mental and neurological illnesses was common among the traditional health practitioners for example when asked about the common terms used to describe epilepsy Kibao said:

‘*You will see them foaming from their mouths*, *you will see their legs twitching*. *That is the beginning*.*’*

Table 1 summarizes the findings on the local terms used for the disorders.

**Table 1:** Local terms for common mental disorders.

Depression	Psychoses	Epilepsy	Child and adolescent mental and behavioural disorders	Disorders due to substance use	Dementia	Self-harm/suicide
**Traditional healers**						
■*Shulamoyo* ^[Kigiryama langauge]^ (self-denial)	■*Wazimu* ^[Kiswahili language]^ (madness)	■*Pepo* ^[Kiswahili language]^ (evil spirit)	■*Nyuni* ^[Kigiryama langauge]^ *(fits)*	■ *Ulevi* ^[Kiswahili language]^ Drunkardness)	No local terms	■*Kujiua* ^[Kiswahili language]^ (killing oneself)
■*Moyo wa dzulu dzulu* ^[Kigiryama langauge]^ (restlessness)		■*Nyago* ^[Kigiryama langauge]^ (fits)	■*Akili yake hayuko sawa* ^[Kiswahili language]^ (their brains are not ok)			■*Kujitia kitanzi* ^[Kiswahili language]^ (loosely translated to ‘putting a loop around oneself’ but commonly used to mean committing suicide’)
		■*Pepopunda* ^[Kiswahili language]^ (tetanus)				
**Health care providers**						
■Huzini nyingi ^[Kiswahili language]^	■*Amerukwa na akili* ^[Kiswahili language]^ they are mad)	■*Kifafa* ^[Kiswahili language]^ (epilepsy)	No data available	■*Maunga* ^[Kiswahili language]^ *(*people who abuse substances such as heroin and cocaine)	No local terms	No data available
	■*Ana kichaa* ^[Kiswahili language]^ (they are crazy)	■*Kufitika* ^[Kiswahili language]^ (convulsions)				
	■*Ana Vilalu* ^[Kigiryama langauge]^ (madness)					
	■*Homa imepanda kwa kichwa* ^[Kiswahili language]^ (Flu has gotten to the head)					
	■*Kushikika* ^[Kiswahili language]^ (loosely translated to mean ‘the person is held up-’, this term is used to describe patients in an acute episode of psychosis)					

### Perceived causes of mental and neurological disorders

The causes of mental illnesses ranged from biological causes to supernatural causes and this was consistent in all the groups. Biological causes ranged from structural or functional abnormalities of the brain which were either congenitally acquired (e.g. epilepsy) or were secondary to other organic causes such as infections and substance use (e.g. psychosis). Supernatural causes such as possession with evil spirits were mostly ascribed to illnesses such as psychosis, dementia and epilepsy whose symptoms included overt manifestations e.g. convulsions in epilepsy, forgetfulness and confusion for dementia, or unkempt appearance and marked behavioral changes such as aggressive behavior in psychosis. Some could tell that conditions like psychoses have their onset in adulthood but not childhood. Matata a traditional healer described the symptoms of psychosis as follows:

‘*The illness of madness has many causes and it does not occur in children*, *it occurs in adults and its occurrence has various reasons*, *there is having demons cast on you maybe because of stealing*, *others smoke bhang and become mad*, *others are bewitched*, *there is no single cause of madness*.*’*

Primary health care providers were aware of other potential organic causes of some psychotic symptoms. For instance, differentials for auditory hallucinations included hyperglycaemia, hearing impairment or side effects of medications such as antiretroviral therapy. Maua, 26-year-old primary care nurse said:

‘*When someone is hyperglycaemic they can also hallucinate*.*’*

There was consensus between traditional health practitioners and health care providers about some causes of mental illness such as infections in psychosis. However, unlike the traditional healers, the health care providers did not acknowledge supernatural causes (e.g. witchcraft) as personal beliefs of causes of mental illness, but as beliefs of members in the community. For some conditions such as epilepsy and psychosis, the traditional health practitioners described a binary classification of the condition into *‘ile ya kawaida’* meaning ‘that which is ordinary’ and *‘ile ambayo si ya kawaida’* meaning ‘that which is not ordinary’ based on the causes and symptoms. According to the traditional healers, this classification was helpful in determining the appropriate forms of treatment. The excerpts below depict descriptions of epilepsy and psychosis respectively from two traditional healers.

‘*In ordinary epilepsy*, *when the patient falls*, *they must urinate or defecate*, *but if you see they have fallen and there are no other signs then that is not ordinary epilepsy*.*’* (Majani)

‘*It is madness but it’s not due to bewitching*. *I want to add that we call those good spirits’* (Taabu)

Table 2 summarizes the perceived causes of each mental illness and suicide.

**Table 2:** Causes of mental disorders.

Depression	Psychoses	Epilepsy	Child and adolescent mental and behavioral disorders	Dementia	Disorders due to substance use	Self-harm/suicide	Dementia
**Traditional healers**							
■Loss of loved ones	■Evil spirits	■Possession by spirits	■Bewitching	■Reading too much	■ Desire to achieve a ‘high’	■Psychosis	No known causes
	■Bewitching		■Secondary bang smoking	■Being possessed			
	■Stress		■Brain disorder	■Untreated infections (malaria)			
	■Demons		■Mosquitoes				
	■Drug and substance abuse			■Brain atrophy			
	■Malaria						
**Health care providers**							
■ An overworked brain	■ Side effects of medication such as anti-retroviral drugs, antimalarial drugs and anti-cancer drugs	-	■Mother spoiling the child	-	-	■Chronic illnesses	No known causes
	■Witchcraft		■Poor upbringing			■Depression	
	■Hallucinations		■Inheriting the behavior from a relative (genetic factors)				
	■Occupational hazards such as inhaling petroleum						
	■Hearing problems						
	■Hypoglycemia						

### Common presentations of mental disorders

There were some similarities in the description of the symptoms of depression in both the traditional health practitioners and health care providers. For instance, both groups described patients as being isolated and appearing worried all the time. Mzee Mambo, a traditional healer, said:

‘*They cannot sit together with many people like we are sitting here*. *They isolate themselves*.*’*

Mazao, a public health specialist described symptoms of patients with depression as follows:

‘*Those who are depressed are just withdrawn from the normal society*, *they just isolate themselves*, *thinking a lot*.*’*

The participants reported that symptoms of psychosis include hallucinations, paranoia and speaking to oneself. One traditional healer described a patient experiencing visual hallucinations as follows:

‘*I will not be seeing anybody*, *but the person [referring to a patient] in their heads and their eyes*, *they see a person*.*’*

In describing lack of insight in people with mental illness, one nurse said:

‘*When a mad person comes*, *they don’t know themselves*, *they must be brought in by someone; they cannot take themselves to hospital*.*’*

Other symptoms included aggressive behaviour, unkempt appearance and depressive symptoms as depicted by the excerpts below from health care providers.

‘*Someone has aggressive behaviours; they can be violent or others can be in a depressive manner*. *They are violent*.*’* (Katana)

‘*The hair was very shaggy*. *Even the nails; she had refused to cut the nails*.*’* (Kingi)

The traditional health practitioners described epilepsy using a cluster of symptoms, which included foaming at the mouth, bruxism, twitching of the legs, and immunity to fevers in childhood. According to health care providers seizures are both a symptom and a synonym for epilepsy. Symptoms of epilepsy according to health care providers included fits, loss of consciousness and excess salivation. According to traditional healers, there are two types of epilepsy, ordinary epilepsy (which is sometimes called ‘the good epilepsy’) and epilepsy which is not ordinary as described in the excerpts by traditional health practitioners below:

‘*In ordinary epilepsy*, *when the patient falls*, *they must urinate or defecate but if you see they have fallen and there are no other signs then that is not ordinary epilepsy*.*’* (Matunda)

‘*Epilepsy which is not ordinary is that of bewitching*.*’ (*Ayubu)

In both types of epilepsy, the patient has seizures but in the latter, the patient does not urinate/defecate after the seizure. Ordinary epilepsy is believed to be congenital while the latter is believed to be caused by bewitching as shown by the quote below from Mwinyi, a middle aged female traditional healer:

‘*The good epilepsy is that one which a child is born and raised with*. *This one is different from one where you are bewitched*. *They are both epilepsy but the one which you are bewitched is excessive*.*’*

The symptoms of children with behavioural and emotional problems included being restless, noisy, getting involved in brawls, beating other children and selfish behaviour. However, these problems were not regarded by health care providers as mental illnesses as described by Amani, a health care provider:

‘*I don’t think it’s a mental condition*.*’ ‘Its poor parenting*.*’*

Alcohol abuse was perceived by both groups as exceeding the daily intake limit which is measured by level of loss of function e.g. ability to perform usual duties. Approximately 75% of the participants said that the daily alcohol intake limit for palm wine, which is the locally available brew, is approximately two bottles where one bottle is approximately 750ml.

‘*You know there is no limit with drinking*, *because you will take two*, *but after the two you now get even more interesting and want to get more*.*’* (Amani)

The limit also depends on many factors such as if someone has eaten prior to drinking and their inherent tolerance level. There are no sex differences in the recommended daily limit. Symptoms of alcohol misuse include erratic behavior and psychosis.

‘*Alcohol in moderation cannot harm you*, *but for me*, *I used to try and moderate my alcohol then I started to feel like drinking more and more and every time I drunk more*, *I felt happier until finally I exploded like a mad man*.*’* (Mambo, traditional healer, 59 years old)

The main symptoms of patients reported to be abusing hard drugs such as heroin and cocaine include sluggish speech, appearing drowsy, being unkempt and, for those suffering from withdrawal, symptoms include confusion as described by two health care providers below.

‘*There are times that when they get all the drugs and they have issues about the withdrawal symptoms they get confused’* (Ruth, nurse,36 years old)

‘*They always look sleepy’* (Katana, nurse 37 years old)

Table 3 summarizes the findings of the common presentations of the priority mental illnesses.

**Table 3:** Symptoms of common mental disorders.

Depression	Psychoses	Epilepsy	Child and adolescent mental and behavioral disorders	Disorders due to substance use	Dementia
**Traditional healers**					
■*Nikukala moyo wasi wasi* ^[Kigiryama language]^ (being worried all the time)	■Speaking to oneself	■Jaundice	■Beating other children	■Psychotic behavior	
■*Kukala kana raha* ^Kigiryama language]^ (as being unhappy)	■Abusive language	■Urinating or defecating then seizure ends (in normal epilepsy)	■ Doesn’t care about others	■ Odd language	
■*Wasiwasi mwingi* ^Kiswahili language]^ (excess worry)	■Hallucinations	■Bruxism		■ Look half dead	
■Isolating themselves	■Paranoia	■Tongue chewing		■ Look possessed	
■Being emotionally unstable	■Lack of insight	■Convulsions			
■Reduced activities, quietness, and expressions of not feeling loved or wanted.					
**Health care providers**					
■Thinking a lot more than usual	■Aggressive behaviors	■Fits	■Being jumpy	■ Sluggish speech	■Forgetfulness
■Hanging themselves if not treated soon	■Being violent	■*Amepoteza Fahamu* ^Kiswahili language]^ (Loss of consciousness)	■Restlessness	■ Unkempt	■They have no orientation to place
■Harming other people	■ Unkempt appearance	■*Ametoa* *Mate* ^[Kiswahili language]^ (salivary frothing)	■Moves all over	■ Withdrawal symptoms such as confusion.	■They hear voices in their heads
■Feeling isolated		■*Amepata* *mnyama wa Juu [*^Kiswahili language]^ (an expression which is used to describe generalized tonic clonic seizures)		■ Always look sleepy	
■ Losing focus at work				■No control	
■Being isolated				■ Cannot think without drinking	
■ Withdrawn from society					
■Being isolated					
■Refusing to eat					
■Sleep disturbance					
■Not moving from where they are the whole day and not talking as easily as they were talking (psychomotor retardation)					
■Daily activities are affected					
■Poor concentration					
■Feeling unwanted					

### Management of mental and neurological disorders

Participants described the methods used to manage different MNSD, as well as the barriers they encountered in the management of these disorders. Traditional health practitioners managed the disorders depending on the attributed cause and on whether the required treatment modality is within their scope. For example, ‘ordinary epilepsy’ which was believed to be congenital was managed using herbs whereas epilepsy that was not ordinary, believed to be caused by demon possession, was managed using concoctions.

‘*For epilepsy which is not ordinary*, *we usually use traditional medicine*. *There are some herbs which we get from the forests*. *But for the other epilepsy*, *we use zebra hooves*. *That is what can eliminate it completely*.*’ (Majani*, *56 years old)*

One traditional healer also stated that they did not manage alcohol and substance use disorders because according to them these were problems for the youth.

Among the traditional healers, there was no consensus on the specific methods of managing disorders. Management was dependent on practical issues such as availability of materials for instance in epilepsy, some traditional health practitioners did not agree with the concoctions used by others because they suggested materials were not available in their settings. Mazuri, a 71-year-old male traditional healer stated that:

‘*Where I come from some people have never even seen the zebras you are talking about*. *So I cannot agree with him or dispute because where I come from that kind of treatment does not exist*.*’*

The health care providers either managed patients at the facilities e.g. patients with depression, referred patients with mental illnesses to tertiary facilities e.g. for first episode psychosis or did not attend to them at all as in the case of alcohol abuse. They were also involved in routine administration of prescription medication for patients with a known diagnosis. For some conditions such as alcohol abuse, management ranged from no intervention at all to psychological interventions such as counselling as described by two health care providers:

‘*We just leave them for it is their habit*.*’* (Sidi, 41 years old)

‘*So we talked with him and called the auntie so that we can have counselling*.*’* (Halua, 31 years old)

A health care provider described the management of depression as follows:

‘*So*, *for that case we just gave him sedatives at least to sleep and relax*, *we gave diazepam*.*’* (Maua, 36 years old)

Regarding prescription of medication to manage MNSD, the nurses were only allowed to prescribe what they referred to as light sedatives such as diazepam. Any other prescriptions were made by doctors. In describing prescription patterns in primary care facilities, one nurse said:

‘*I just know that the drugs that you are supposed to prescribe are the very light sedatives like the phenobarbital*, *diazepam but anything apart from that we leave for the seniors [referring to doctors]*.*’* (Halua, 31 years old)

Lack of drugs was also a hindrance to prescription by the nurses as described by one nurse working in a remote primary care facility:

‘*We just don’t have the drugs*.*’* (Karim, 37 years old)

Health care providers preferred to refer suspected cases of psychiatric patients even without examining them adequately. One nurse for instance said that patients were referred directly to tertiary facilities, in this case Port Reitz Hospital, which is the only public stand-alone mental hospital serving Kilifi county.

The commonest reason for this included lack of confidence in their ability to handle psychiatric patients due to lack of adequate practical exposure during their training as described by one nurse below:

*‘We did a lot of theory and then we were attached for a practical for two weeks and that was it*.*’* (Mshale, 31 years old)

However, the health care providers showed willingness to undertake refresher trainings on the management of MNSD if provided with management algorithms.

‘*All of us undertook psychiatric training in the colleges but it is rusting because we have not been practicing it*, *so if we go for a refresher training we refresh what we were trained then we are given the job*, *we can do it*.*’ (Nadia*, *39 years old)*

### Collaboration between biomedical service providers and traditional healers

Traditional health practitioners were willing to collaborate with health care providers and they routinely referred patients who they could not manage as described by two traditional healers:

‘*When they first come*, *I thoroughly examine them whether they have a mental illness or what*. *I must do a thorough examination and then if I cannot manage them I must send them to the hospital*.*’* (Kizito,69 years old.)

Referring to health care providers, one traditional healer said:

‘*I support them because I will have tried my ways and applied my expertise and once I am done*, *I must also invite the doctor*.*’* (Tundo,49 years old)

The traditional health practitioners cited reasons for not being able to manage some patients as non-response to traditional medicine.

‘*I will give you my medication*, *and that medication I will give you for 3 days*, *on the fourth day if there is no improvement then you have to go to hospital*.*’* (Makao, 52 years old)

According to health care providers, traditional health practitioners were the first point of care of patients with mental illnesses. Tende, a 29-year-old nurse said:

‘*When you tell somebody that they are suffering from maybe depression*, *they will not accept it*, *in fact when you refer them to a facility they say they are going to try the traditional medication then there after they go to Port Reitz [a tertiary standalone mental health hospital located in the neighbouring Mombasa county]*.

Health care providers were not willing to collaborate with the traditional healers. In their view, traditional health practitioners worsened the prognosis of patients with mental illness. Jana, a 46-year-old nurse said:

‘*Instead of seeking treatment they go to witchdoctors*, *the condition worsens more than even if they were brought to the doctor*.*’*

Health care providers also viewed taking patients with mental illness to traditional health practitioners is a violation of the patients’ human rights mainly because it denied them early medical treatment and lead to a deterioration of the patient’s state as described by one health care provider:

‘*They go there and the things there are not good*, *they[referring to traditional healers] bring you a patient when in bad condition*.*’ (*Zuri, 33 years old)

## Discussion

To the best of our knowledge, this is the first study that examined the perspectives of health care providers and traditional health practitioners in all the 7-priority mental and neurological conditions listed in the mhGAP-IG. A recent South African study which examined the role of traditional health practitioners in the management of MNSD, overemphasized on seizure disorders at the expense of other MNSD[24] Few studies have focused on perspectives of both traditional health practitioners and primary health care workers in one study but no study has examined all the priority MNSD in one study. For examples studies evaluating the effectiveness of task shifting to lay health workers in primary health care system in Zimbabwe mainly focused on depression and did not include traditional healers[25] Our results show that both traditional health practitioners and primary health care providers have shared and divergent views about MNSD and their management, which will inform the design of interventions for management of MNSD in this setting. It also identifies MNSD areas requiring future attention. Understanding the local terms and perceived causes used in the community also will help the primary health care providers to understand the diagnosis to work with when receiving MNSD referrals from the community or from traditional healers.

Although our study focused on the biomedical and alternative medicine models, our findings were like those of previous studies which showed that care for MNSD is complicated and often shifts between these two models.

### Recognition and terms of MNSD

Local terms of priority MNSD are helpful in providing perspective about perceived causes and therefore the treatment options offered to people with MNSD. For instance, one local name for epilepsy is “nyuni” which according to previous studies conducted in this setting represents a specific type of epilepsy caused by spirits whose only management is exorcism by the traditional health practitioners (10). Additionally, a requirement of the implementation process of the mhGAP is the contextualization to make it locally relevant. Local terms will therefore help contextualize the guidelines to make the priority conditions easily understandable.

Psychosis was the only condition for which most of the common presenting features listed in the mhGAP-IG were described by both groups, suggesting that this is a well-recognized condition in this rural community. Both the traditional health practitioners and the primary health care providers also described psychosis as the most common mental disorder in their practice. This finding is consistent with that of a recent situation analysis of the state of mental health services in this setting, which found that over 60% of psychiatric patients visiting outpatient facilities were diagnosed with psychosis [22]. This could be because of the overt manifestations of the symptoms of acute psychosis which are very disabling thus easily identifiable. The term ‘madness’ was consistently used by both groups to refer to people with psychosis. Whereas the traditional health practitioners cited using this term in their practice, the health care providers cited this as a term used in the community rather than one that they used themselves, despite the question asking them about their own perspectives. In a study conducted in three LMIC about the concepts of madness, the investigators found similar findings among the health care providers and concluded that this was because of the framing of their questions which were not asking about the participants’ personal views [26]Our study has found contrasting results in that even when the questions are framed to ask about the participants’ personal views, the health care providers present the use of derogatory terms as those of the community and not their own. This finding might be a consequence of the limitations posed by a focus group discussion as a method of data collection where respondents tend to give socially acceptable answers. Neurological conditions were often classified as mental illnesses probably because of shared beliefs about causes particularly supernatural powers, which are often believed to be controlling the mind. The perception of neurological disorders as mental illnesses may be because the symptoms for these disorders often co-occur in one person, but this should be objectively established through epidemiological studies.

The participants did not recognize some MNSD, suggesting that these conditions may not be common in this setting or that they are poorly detected because perhaps patients do not seek care. Dementia was not commonly recognized in this setting which may inform its exclusion in the implementation phase of the study. This finding is like that found in a study in Nigeria which excluded dementia after the contextualization phase of their mhGAP-IG implementation process (7). This however should not be interpreted to mean that dementia does not exist in this setting and could be because of the very low life expectancy (57.5 years for males and 56.3 year for females)[27] in this setting, which may mean that most people die before the onset of dementia. It could also be because the common symptoms of dementia are considered as a normal part of ageing process and therefore case detection is difficult. The descriptions for epilepsy as a disorder characterized by convulsions but one which can be associated with evil spirits replicates previous studies in this rural area (10). However, tetanus which is an infectious disease, was mentioned synonymously with epilepsy probably because both conditions presented with spasms (i.e. tetanoid spasms and epileptic spasms). This finding shows the need to educate health care workers and traditional health practitioners about the diagnostic and management differences between these conditions. Child and adolescent mental and behavioural disorders were not very commonly encountered by both groups. In fact most clinicians did not consider behavioural and emotional problems as mental disorders but as a result of poor upbringing, in spite of epidemiological evidence showing that the burden of behavioral problems in this setting is substantial[28].These problems were not encountered in hospital perhaps because parents did not think they required biomedical attention. However, the perception of behavioral and emotional problems as problems related with poor upbringing provides a window of opportunity for implementing parental interventions in this rural area.

### Characterization of MNSD

Depression was the only condition that was rightly categorized (according to the Diagnostic and Statistical Manual Version V criteria) as a ‘mood disorder’ by the health care providers who described it as a problem that interferes with patients’ moods. It was also the MNSD with the most detailed description, often defined by features such as low mood, unhappiness, being worried, disturbances in routine activities including sleep. Somatization was not listed as a symptom of depression and this was consistent with a previous study on depression in this setting (11). Despite this detailed description of depression, a previous situation analysis showed that most cases of depression were not treated in hospitals [22]. In that paper the authors hypothesized that this may be due under-recognition of depression in the community but results from our work show that perception of depression as a mild condition not requiring treatment is another plausible explanation. Only positive symptoms for psychosis such as hallucinations, paranoia and abnormal behaviors were appreciated by the traditional health practitioners and health care providers. These positive symptoms are easy to identify and would often result in visits to either traditional health practitioners and health care providers, perhaps explaining why psychosis is the most common MNSD in outpatient facilities in this area. The recognition that people with psychoses do not have insight is important and should be emphasized during awareness campaigns to encourage members of the community to assist the affected people with help seeking. Even if such positive symptoms form the vast burden of psychosis, negative symptoms such as social withdrawal and apathy should not be overlooked.

Epilepsy was extensively described by traditional healers. The better understanding of epilepsy by the traditional health practitioners that is apparent in this study may be because many of the respondents of our study had previously participated in an epilepsy education intervention [8]. From this study the traditional health practitioners gained knowledge on some causes of epilepsy and they may have applied this knowledge in answering the questions for this present study. The features given for epilepsy such as unconsciousness, tongue biting and excess salivation pointed to recognition of the generalized tonic-clonic forms of epilepsy, previously referred to as grand mal seizures. These types of epilepsy are dramatic and easily identifiable by the lay people, but it should be appreciated that focal forms of epilepsy are more common in this area, and their presentations should be emphasized in trainings of traditional health practitioners and health care providers. It was surprising to see jaundice mentioned as one of the manifestations of seizure disorders and could suggest that many seizures occur in the neonatal period when jaundice is common. These findings suggest that circumstances in which seizure disorders occur could be clarified to the members of the community. Descriptions of behavioral disorders included restlessness and conduct problems. This description is20 helpful in detecting attention deficit hyperactivity disorders in the community but emotional problems may be missed. A tool like the Child Behavior Checklist, which has been validated for use in pre-school children in this setting, may be helpful in identifying emotional problems [29]. Substance abuse disorders were well characterized, perhaps because palm wine use and abuse of hard drugs is common on the Kenyan coast. The most encouraging reported feature of substance abuse is the appreciation that there is drug-induced psychosis.

### Management of MNSD and collaboration between healthcare workers and traditional healers

Some health care providers could write prescriptions for psychotropic medications, although they often referred difficult cases. Some admitted that if they were trained on how to make these prescriptions they were willing to manage common MNSD at primary care facilities as a first step, before referring patients to secondary or tertiary facilities. This can be addressed through the implementation of evidence based task sharing approaches such as the mhGAP-IG trainings[14] which equips non-specialized health care providers with skills and algorithms of recognizing and managing common mental disorders.

Traditional health practitioners treated patients with MNSD according to the nature of the problem, with herbs used for disorders with a biological basis e.g. those with a clear onset early in childhood, and concoctions for less understood MNSD that are thought to be caused by evil spirits. There was no consensus among the traditional about the management of some MNSD such as epilepsy. The differences in management practices may be due to the different regions or dialects represented in the study, which may indicate that although the Mijikenda represents one ethnic group, there may be nuances in cultural beliefs and practices across the dialects. It may be worth working with traditional health practitioners to perform some laboratory analysis on the ingredients and mechanisms of actions of the herbs and concoctions used, as some of them may have some therapeutic benefits. Even if these drugs are found to be ineffective, traditional health practitioners can be trained to offer psychosocial support to patients or even to help improve the treatment gap of MNSD.

Although traditional health practitioners were willing to collaborate with health care providers, the reverse was not true for the health care providers. Although the frameworks of how a collaborative arrangement would look like and how it would work is beyond the scope of this study, future studies should explore potential avenues of initiating dialogue between these two groups to encourage the health care providers to appreciate the role of traditional health practitioners in managing MNSD.

The results of this study helped in understanding the local idioms of common MNSD. The study has shown that some of the common MNSD do exist in this setting and are recognized and managed by both health care providers and traditional healers. It has also highlighted the need for further training of the primary health care providers to detect and manage common MNSD. There is also a need to encourage traditional health practitioners to make immediate referrals of patients with MNSD to health facilities to ensure better prognosis. Finally, this study suggests that an effective package of care for MNSD in this setting may be one that includes collaboration between traditional health practitioners and health care providers.

### Study limitations

The study did not assess the level of knowledge or expertise of the primary health care providers in diagnosing and managing MNSD. This may have influenced the responses of the participants. We however assumed that the primary health care providers had at least baseline knowledge, which is offered as part of the training curriculum for nursing and medical courses in Kenya. We did not measure knowledge retention or how frequently this knowledge was translated into practise. Our study did not examine how factors such as the role of the family influenced choice of health care provider and further work is required to examine these factors. Every effort was made to ensure that there was a good balance in the characteristics of the interviewers such as age, sex, level of experience and building a rapport with the participants. However, it is still possible that some factors may have influenced the range of responses. For instance, the primary health care providers may have given socially acceptable answers because of knowledge about the upcoming mhGAP training where one of the interviewers (MB) is a member of the planning team, or because they were aware they were being interviewed by researchers.

## Supporting information

S1 File Interview guides for traditional healers and primary health care providers. (DOCX)
