# Supplementary material for: Priority mental, neurological and substance use disorders in rural Kenya: Traditional health practitioners’ and primary health care workers’ perspectives
Source: PLoS One. 2019 Jul 23;14(7):e0220034. doi: 10.1371/journal.pone.0220034 (PMC6650073; doi:10.1371/journal.pone.0220034)
Supplement: S1 File — (DOCX) [file pone.0220034.s001.docx]

**S1 file: Interview guides for traditional healers and primary health care providers**

The following interview guides are included in this section:

- Focus group discussion guide for both traditional healers and primary health care providers in English, Kiswahili and Kigiryama
- Vignettes for each of the priority conditions in English, Kiswahili and Kigiryama

**Focus group discussion guide [English]**

Introduction: In this discussion, I would like to understand your experiences and views around mental, neurological and substance use disorders. Although there are many types of these illnesses, I will focus on 7 disorders because the World Health Organization has identified them as priority disorders, which means that they require urgent attention when planning for delivery of mental health services. The results of this study will help us to contextualize an intervention called the mental health Gap Action Programme, which will be used here in Kilifi. I would like to get your views on the following areas.

1. The local terms used to describe these illnesses
2. The signs and symptoms of these illnesses
3. How you manage these disorders in your practice

Additionally, research here in Kilifi has shown that some patients chose to seek help from traditional healers and others chose hospitals while some use both services. We are going to ask for your views about collaboration between medical practitioners and traditional healers. There are no right or wrong responses to these questions.

**Topic guide questions**

1. **What are some examples of mental, neurological or substance use disorders?**

- Probe for synonyms for each of the conditions mentioned

Specific questions

How would you describe mental illnesses?

What are some examples of mental illnesses?

How would you describe neurological disorders?

What are some examples of neurological disorders?

How would you describe substance use disorders?

What are some examples of substance use disorders?

1. **What are some of the examples of common signs and symptoms of these illnesses that you have mentioned**

- Probe for signs for each of the conditions mentioned

Specific questions

You have mentioned some conditions above. What are the common signs of [*name each condition*]?

I am now going to read a short description and ask you a few questions

| Read vignette one at a time, and then ask questions 3 and 4 after each vignette |
| --- |

1. **Have you managed/treated a patient with these symptoms?**
2. **[If yes for question 3] How do you manage people with these disorders?**

| - For each of the question, solicit as many responses as possible among respondents - Ask for management for each of the disorders mentioned |
| --- |

1. **In your practice have you collaborated with a medical practitioner/ traditional healer**

Specific questions

[for traditional healers] In your practice, have you referred patients to a medical practitioner?

- If yes, probe further on the circumstance that lead to the referral and the outcome of the referral i.e did they get any feedback from the patient after the referral? Did the medical practitioner try to contact them?
- If no ask: Is there any reason why not? And probe for explanation

[for traditional healers] In your practice, have you received referrals from medical practitioners?

- Repeat probes above

In your view, do you think that traditional healers and health care providers can work together?

- *For any response (yes/no) probe for explanations*

**Focus group discussions [Kiswahili]**

Utangulizi: Katika mjadala huu, ningependa kuelewa maoni yako kuhusu matatizo ya kiakili na ya mishipa mwilini. Ingawa kuna aina nyingi za matatizo haya, tutazingatia matatizo 7 kwa sababu Shirika la afya duniani limetambua matatizo haya kama matatizo ya kipaumbele, ambayo ina maana kwamba tunahitaji kuzipa kupaumbele tunapopanga huduma za afya ya akili.Matokeo ya utafiti huu yatatusaidia Kuboresha mfumu mpya wa mafunzo kwa madaktari unaitwa (mental health Gap Action Program Intervention Guidelines ama mhGAP-IG kwa kifupi. Ningependa kupata maoni yako kuhusu mada zifuatazo

1. Istilahi au maneno yanayotumika kutambua magonjwa haya
2. Dalili za magonjwa haya
3. Matibabu ya magonjwa haya

Utafiti hapa Kilifi umeonyesha kwamba baadhi ya wagonjwa hutafuta matibabu kutoka kwa madaktari wa kiasili, wengine kutoka mahosiptali na wengine kutoka pande zote mbili. Tutakuuliza moni yako kuhusu ushirikiano kati ya madaktari wa hospitalini na matabibu wa kiasili. Hakuna jibu ambalo sio sahihi.

**Vielekezo vya mjadala**

1. **Je, mifano ya matatizo ya kiakili na ya mishipa ya akili na ipi?**

-Uliza kuhusu majina badala katika kila mfano

Maswali

Kwa maoni yako, matatizo ya kiakili ni nini?

Je mifano ya matatizo ya kiakili ni yepi?

Kwa maoni yako, matatizo ya mishipa ya kiakili ni nini?

Je mifano ya matatizo ya mishipa ya kiakili ni yepi?

Kwa maoni yako, matatizo ya matumizi mabaya ya madawa ni nini?

Je mifano ya matatizo ya matumizi mabaya ya madawa yepi?

**2. Je baadhi ya mifano ya dalili za kawaida za magonjwa uliyotaja ni gani?**

- Uliza kuhusu daliili za kila ugonjwa uliotajwa

- Uliza iwapo dalilli hizi lazima zitokee kwa pamoja kabla utambuzi wa ugonjwa ufanywe

Nitakusomea maelezo mafupi kisha nikuulize maswali machache baadaye

| Soma maelezo, moja kwa wakati mmoja,kisha uulize swali nambari3 na 4 baada ya kila aya ya maelezo |
| --- |

**3.Je umewahi kutibu mgonjwa mwenye dalili hizi?**

**4. [Iwapo jibu ni ndio kwa swali 3] Elezea Zaidi kuhusu matibabu unayowapa wagonjwa**

| - Ulizia majibu nyingi iwezekanavyo - Uliza swali kwa kila ugonjwa uliotajwa |
| --- |

**5.Katika utendaji kazi wako, je umewahi kushirikiana na daktarin/tabibu wa kiasili?**

Maswali

[matabibu wa kiasili ] Je katika utendaji kazi wako, umewahi kuwatuma wagonjwa kwa madaktari hospitalini?

- Ikiwa jibu ni ndio, uliza Zaidi kuhusu mambo yaliyowapelekea kuwatuma wagonjwa hospitalini. Kwa mfano, je walipata majibu yoyote kutoka kwa wagonjwa wao baada ya kwenda hospitalini? Je madaktari walijaribu kuwasiliana nao, na kadhalika.
- Ikiwa jibu ni la, ulizia sababu pamoja na mifano ikiwa inawezekana.

[matabibu wa kiasili] Je katika utendaji kazi wako, umewahi kupata wagonjwa waliotumwa kwako na madaktari?

- Rejelea maswali uliyouliza hapo juu

Kwa maoni yako, unadhani kuwa matabibu wa kiasili wanapaswa kushirikiana na madakitari?

- *Kwa jibu lolote, ulizia mifano na maelezo*

**VIGNETTES**

**Depression**

*Halima is a 46 year old woman whose husband died a few months ago. She was left with 4 children to take care of and she has no stable source of income and no support from her family. She had started experiencing poor sleep and loss of appetite soon after her husband died. The symptoms worsened after the funeral. She started experiencing aches all over her body which led her to consult the local clinician. There she was told she is well was prescribed sleeping pills and vitamins. Although her sleep improved immediately, the situation worsened again and she went back to the clinic and was given more sleeping pills and injections. This went on for months until she could no longer sleep without the pills.*

**Psychosis**

*“Gambo, a 20-year-old university student, was brought* [to traditional healer/ primary health care provider] *because she locks herself in her room. Gambo used to be a good student but has failed her last* *exams. Her mother said that she would often spend hours staring into space. Sometimes she appeared to say things to herself in a low and inaudible voice as if she were talking to an imaginary person. Gambo was forced to come to the clinic/traditional healer by her parents. At first, she refused to talk to the nurse/**traditional healer. After a while she admitted that she believed her parents and neighbors were plotting to kill her and that the devil was interfering with her mind. She also said that she did not see why she had been brought to the hospital/traditional healer.”*

**Dementia**

*Randu was a 75 year old retired teacher living with his two sons and their wives. Over the past few years, Randu became increasingly forgetful but his family just passed it off as “growing old”. However, the forgetfulness grew worse until one day he lost his way around the compound. He started forgetting names of his relatives including his grandchildren. His behavior became unpredictable; on some days he would sit for hours without saying anything while on other days he would easily lose his temper and become irritable. Randu’s physical health began to deteriorate and one day he had a fit. He was taken to hospital and a scan on the brain showed that there were changes in the structure of the brain.*

**Epilepsy**

Mcheshi is a 21-year-old housewife and mother of two. A few months ago, while cooking for her family, she experienced fits which made her fall near the fireplace. She lost consciousness and when she regained it, she realised that she had sustained burn injuries on her arms and legs. She could not remember what happened before the fits and she complained of a severe headache and muscle pains. This was the second time that Mchesi had had fits. The first episode occurred last year. Prior to this first episode, Mchesi did not have any illness and she was not involved in any accident.

**Suicide and self-harm**

*Mambo is a 40-year-old man fisherman who lives with his ageing parents in the village. Mambo also takes care of his two children after his wife died 2 years ago from an unknown illness. In the past 3 months, Mambo’s fishing business deteriorated since he spent a lot of time at home taking care of his parents and children. Mambo depleted his savings and was unable to get any other employment. Mambo felt like life was meaningless and that he had no purpose to live. Mambo started telling his friends and family goodbye, without explaining where he was going and he also started giving away his little possessions. Mambo also asked his sister to take care of his children when he was gone.*

**Disorders due to substance use**

1. **Alcohol use disorder**

*Mshale is a 30-year-old construction worker. He is married with three children. Over the past six months, Mshale has taken over 5 sick leaves from work. He has been getting stomach upsets and even developed jaundice. Mshale is worried about his health because he must drink alcohol before he can start his day and before having lunch and he says that no matter how much he drinks, it never feels enough. What frightens him the most is that he wakes up feeling terrible.*

1. **Drug and substance use (e.g heroin, cocaine, bhang, cannabis)**

*Dada is a 16-year-old high school student. She has always been an average student, hardworking and honest. Recently however, her mother noticed that Dada had been staying late at night, her school grades had been falling and she was spending more money. The previous week, her mother noticed that money was missing from her purse. She was worried that Dada might have stolen it. Dada was no longer spending time with her old friends and was hanging around a new group of friends whom she had not introduced to her mother. Her mother suggested that they see a counsellor, which Dada refused. The health worker decided to visit Dada at home. At first Dada was reluctant to talk to the counsellor. After several months, she admitted that she was “hooked” and she had tried to stop on many occasions but each time she tried, she felt so sick that she just went back to the drug.*

**Child and adolescent mental and behavioural problems (CAMHP)**

1. **ADHD**

*Nzaro is a seven-year-old boy studying at a local primary school. His performance at school is below average. His teacher notified his parents that during lessons, Nzaro moves from one desk to another and disrupts other students. At home Nzaro cannot complete a task and is always jumping on the seats and on the bed.*

1. **Autism**

M*arya is a 6 year of girl. She is the youngest of 3; her sisters are aged 8 and 11. Marya’s mother noticed that Marya does not like playing with her siblings or with other children of her age. Marya plays by herself and does not like group plays. Marya also gets very angry her siblings take a different route to school other than the one she is used to. Marya’s mother also observed that Marya does not respond to facial expressions such as smiles or frowns.*

| *If participants had names any other CAMHP such as internalising problems or externalising problems, ask questions 3 and 4 based on their examples* |
| --- |

**Maelezo mafupi**

**Sonona**

Halima ni mwanamke wa umri wa miaka 46 ambaye mumewe alikufa miezi michache iliyopita. Aliachwa na watoto wanne, na hana mapato wala hapati msaada wowote kutoka kwa familia yake. Halima alianza kuwa na matatizo ya usingizi na kukosa hamu ya chakula punde tu baada ya mumewe kufariki. Dalili hizi zilikithiri baada ya mazishi. Alianza kuhisi maumivu kote mwilini na ikampelekea kwenda hospitalini. Hospitalini, aliambiwa kuwa hana tatizo lolote, akapewa dawa za kuongeza usingizi na vitamini. Ingawaje usingizi wake uliimarika, hali yake ilirudia kuwa mbaya Zaidi baaday yam da mchache. Aliporudi hospitalini, alidungwa sindano na akaongezewa dawa za kulala. Hali hii iliendelea kwa miezi mpaka hakuweza tena kulala bila ya dawa.

**Tatizo sugu la kiakili**

"Gambo, mwanafunzi wa Chuo Kikuu mwenye umri wa miaka 20, aliletwa [kwa tabibu wa kiasili/ mhudumu wa afya]kwa sababu yeye hujifungia chumbani mwake. Gambo alikuwa mwanafunzi mwadilifu lakini alianguka mtihani wake wa mwisho. Mama yake alisema kwamba yeye alitumia muda mwingi kuduwaa. Wakati mwingine Yeye alionekana kusema mambo mwenyewe kwa sauti ya chini kana kwamba anazungumza na mtu asiyeonekana. Wazazi wake Gambo walimlazimisha kwenda kwa mhudumu wa afya/tabibu wa kiasili. Mara ya kwanza, alisita kumzungumzia mhudumu wa afya/tabibu wa kiasili. Baadaye, alikiri kwamba aliamini kuwa wazazi wake na jirani zake walikuwa wanapanga kumuua na kuwa shetani alikuwa anaingilia fikra zake. Pia hakuwelewa kwanni alikuwa ameletwa kupata matibabu.

**Tatizo la kiakili katika uzee**

Randu alikuwa mwalimu mstaafu mwenye umri wa miaka 75, aliyeishi na wanawe wa kiume wawili pamoja na wakaza wanawe. Katika miaka michache iliyopita, Randu akawa inazidi kusahau lakini familia yake tu ilidhani kuwa ni “kuzeeka tu kwa kawaida”. Hata hivyo, usahaulifu na ilikua mbaya zaidi hadi siku moja alipoteza njia kwenye boma lake. Alianza kusahau majina ya ndugu zake ikiwa ni pamoja na wajukuu wake. Tabia yake akawa haitabiriki; siku nyingine alikaa kwa masaa bila kusema chochote na wakati mwingine alikereka kwa haraka na kuwa mwepesi wa hasira. Afya ya kimwili ya Randu ikaanza kuzorota na siku moja alifitika. Alipelekwa hospitali na tambazo kwenye ubongo ilionyesha kwamba kulikuwa na mabadiliko katika muundo wa ubongo.

**Kifafa**

Mcheshi ni mwanamke mwenye umri wa miaka 21, na pia ni mama wa watoto wawili. Miezi kadhaa iliyopita alifitika alipokuwa anapikia jamii yake. Kufitika huku kuliwamngusha karibu na moto. Alipoeza fahamu na fahamu zilipomjia, aligundua kwamba alikuwa amechomeka kwenye mikono na miguu yake. Hakuwa na kumbukumbu yoyote ya yaliyojiri kabla ya kufitika. Alilalamikia kuhusu maumivu makali ya kichwa nay a misuli. Hii ilikuwa mara ya pili ya tukio hili kutokea. Mara ya kwanza ya Mcheshi kufitika ilikuwa ni mwaka uliopita. Kabla ya tukio la kwanza la kufitika, Mcheshi hakuwa na ugonjwa wowote na hakuwa kwenye ajali yoyote.

**Kujidhuru au kujiua**

Mambo ni mvuvi mwenye umri wa miaka 40 mtu ambaye anaishi na wazazi wake kijijini. Mambo pia ndiye mlezi wa watoto wake wawili baada ya mke wake kufariki miaka 2 iliyopita kutokana na ugonjwa usiojulikana. Katika miezi 3 iliyopita, biashara ya uvuvi wa Mambo ilisambaratika kwani alitumia muda mwingi kuwatunza wazazi wake nyumbani na wanawe. Akiba aliyowekeza mambo ilifilisika na hakuweza kupata ajira nyingine yoyote. Mambo alihisi maisha hayakuwa maana na kwamba hakuwa na kusudi la kuishi. Mambo alianza kuwaambia marafiki zake na familia kwaheri, bila kueleza ambapo alikuwa anakwenda na pia alianza kugawa mali yake. Mambo pia alimwomba dada yake awatunze watoto wake atakapokuwa ameenda.

**Matatizo yanayotokana na matumizi ya madawa ya kulevya au pombe**

1. **Matumizi mabaya ya pombe**

Mshale ni mfanyakazi wa ujenzi mwenye umri wa miaka 30. Yeye ameoa na ana watoto watatu. Katika miezi sita iliyopita Mshale aliomba ruhusa kazini Zaidi ya mara 5 kwa sababu ya ugonjwa. Amekuwa na matatizo ya mutmbo na pia ana homa ya manjano. Mshale ana wasiwasi kuhusu afya yake kwa sababu lazima anywe pombe kabla ya yeye kuanza siku zake na kabla ya kuwa na chakula cha mchana na anasema kwamba haijalishi ni kiasi gani atakunywa, kamwe hatosheki. Kinachomtisha Zaidi ni kwamba yaya huamka akihisi vibaya mwilini.

1. **Matumizi ya madawa ya kulevya**

Dada ni mwanafunzi wa shule ya sekondari ya mwenye umri wa miaka 16. Daima amekuwa mwanafunzi wa wastani, anayefanya kazi kwa bidii na uaminifu. Hizi maajuzi, mamake Dada amemwona akikaa nje sana usiku, alama zake za shuleni zinapungua na anatumia pesa Zaidi. Wiki iliyopita, mamake Dada alipoteza pesa kwenye mkoba wake na akahofia kuwa Dada alizichukua pesa hizo. Dada alikuwa hatangamani tena na Rafiki zake wa zamani, na alikuwa ana kuzungukazunguka na kikundi kipya cha marafiki amabo hakuwatambulisha kwa mama yake. Mama yake alipendekeza kwamba wakamwone mshauri wa afya, na Dada alikataa. Mhudumu wa afya aliamua kutembelea Dada nyumbani. Hapo mwanzoni, dada alisita kuzungumza na mshauri. Baada ya miezi kadhaa, yeye alikiri kwamba yeye alikuwa “ameshikwa mateka” nay a kwamba kila alipojaribu kujinasua, alihisi mgonjwa sana, ikambidi arudie madawa.

**Matatizo ya afya ya kiakili ya watoto na vijana**

1. **Kutotulia**

Nzaro ni mvulana mwenye umri wa miaka saba anayesoma katika shule ya msingi. Utendaji wake shuleni ni chini ya wastani. Mwalimu wake alimjulisha wazazi wake Nzaro kwamba wakati wa masomo, Nzaro hutua kutoka dawati moja hadi nyingine na kuwatatiza wanafunzi wenzake. Nyumbani Nzaro hawezi kukamilisha kazi na daima yeye kuruka juu ya viti na juu ya vitanda.

1. ***“Autism”***

Marya ni msichana mwenye umri wa miaka 6. Ana ndugu wawili, mmoja wa miaka 8 na mwingine wa miaka 11. Mamake Marya aliona kwamba Marya hapendi kuheza na watoto wengine wenye umri wake, au hata na ndugu zake. Marya hupenda kucheza peke yake na hapendi kucheza kwa vikundi. Marya pia hukasirika sana kila ndugu zake wanapobadilisha nji yao ya kwenda shuleni. Mama wa Marya pia aliona kwamba Marya haikubali imisho za uso kama vile tabasamu.
